# Effect and mechanical mechanism of spontaneous breathing on oxygenation and lung injury in mild or moderate animal ARDS

**DOI:** 10.1186/s12890-023-02730-y

**Published:** 2023-11-04

**Authors:** Rui Yang, Leilei Zhou, Zongyu Chen, Shuang He, Siyu Lian, Yi Shen, Xianming Zhang

**Affiliations:** 1 First People’s Hospital of Guiyang City, Guiyang, Guizhou, China; 2 Department of Respiratory Medicine, The Affiliated Hospital of Guizhou Medical, 28 Guiyi Street, Guiyang, Guizhou, 550000 China

**Keywords:** Acute Respiratory Distress Syndrome (ARDS), Spontaneous Breathing (SB), Complete muscle paralysis(PC), Beagle dogs, End-Expiratory Lung Volume (EELV)

## Abstract

**Objective:**

The present study aimed to determine the effect and mechanical mechanism of spontaneous breathing during mechanical ventilation on oxygenation and lung injury using Beagles dogs mild or moderate acute respiratory distress syndrome (ARDS) model.

**Methods:**

After inducing mild or moderate ARDS by infusion of oleic acid, Eighteen Beagles dogs were randomly split into Spontaneous breathing group (BIPAP_SB_, *n* = 6), and Complete muscle paralysis group (BIPAP_PC_, *n* = 6),Six Beagles without ventilator support comprised the control group. Both groups were ventilated for 8 h under BIPAP mode. High-pressure was titrated TV to 6 ml/kg. A multi-pair esophageal balloon electrode catheter was used to measure respiratory mechanics and electromyogram. End-expiratory lung volume (EELV), gas exchange and respiratory variables were recorded in the process of mechanical ventilation. The contents of Interleukin (IL)-6 and IL-8 in lung tissue were measure using qRT-PCR. Besides, lung injury score was calculated in the end of mechanical ventilation.

**Results:**

Based on the comparable setting of ventilator, BIPAP_SB_ group exhibited higher safety peak transpulmonary pressure, abdominal pressure, EELV and P/F(PaO2/FiO2) than BIPAP_PC_ group, whereas mean transpulmonary pressure, the mRNA levels of the IL-6 and IL-8 in the lung tissues and lung injury score in BIPAP_SB_ group were lower than those in BIPAP_PC_ group.

**Conclusion:**

In mild to moderate ARDS animal models, during mechanical ventilation, SB may improve respiratory function and reduce ventilator-induced lung injury. The mechanism may be that spontaneous inspiration up-regulates peak transpulmonary pressure and EELV; Spontaneous expiration decreases mean transpulmonary pressure by up-regulating intra-abdominal pressure, thereby reducing stress and strain.

## Introduction

Acute respiratory distress syndrome (ARDS) is common in critically ill patients admitted to intensive care units. Besides, the major supportive therapy for this syndrome is mechanical ventilation [1]. However, mechanical ventilation has side-effects, and it is likely to induce P-SILI (patient self-inflicted lung injury) and ventilator-induced lung injury (VILI) [2]. Despite the wide use of lung protective ventilation strategies [3], the overall intensive care units and hospital mortality of ARDS patients remain above 40% [4, 5].

Spontaneous breathing(SB) and Complete muscle paralysis(PC) are two auxiliary treatments for ARDS.Among them, SB is divided into assisted SB and unassisted SB according to the presence or absence of mechanical assistance. Completely controlled ventilation is primarily with the use of neuromuscular blocking agents [6]. During mechanical ventilation in patients with ARDS, however, the role of SB is contradictory [7]. Several studies reported that spontaneous breathing followed by a strong SB effort can induce lower pleural pressure, high transpulmonary pressure and rapid respiratory rate (RR), thereby up-regulating intrathoracic blood volume, worsening pulmonary edema, and then increasing lung damage, may induce patient self-inflicted lung injury（P-SILI）and ventilator-induced lung injury (VILI) [8]. However, numerous experimental and clinical studies also reported that SB with activity of the inspiratory muscles can induce greater pleural pressures and transpulmonary pressure, thus facilitating the homogenous distribution of ventilation, diminishing atelectasis [9, 10], and further reducing lung mechanical stress and strain [11, 12]. Recently, Marcelo et al. found that in animal with severe ARDS, SB could aggravate lung injury; in animal with mild or moderate ARDS, SB might be more protective for injured lung, whereas the precise mechanism is unclear [13]. Our previous studies have also confirmed that SB aggravates lung injury in severe ARDS animal models, and the mechanism may be related to abdominal muscle activity [8], however, the role and mechanism of spontaneous breathing in mild to moderate ARDS has not been fully elucidated. In mild to moderate ARDS, the role of abdominal muscle activity in mechanical ventilation is not clear. In this study, we explored the effect of spontaneous breathing on lung tissue and its mechanism in oleic acid-induced mild to moderate ARDS animal model, in order to provide theoretical basis for the treatment of mild to moderate ARDS.

## Method and material

This study was approved by the ethics committee of Guizhou medical university. The care, and handling of the animals were in compliance with the National Institutes of Health Guidelines for the Care and Use of Laboratory Animals standard.

### Preparation of animal samples

A total of 18 male beagle dogs were taken in this study. The weights ranged from 9.5 to 12.8 kg. The animals were pre-medicated with ketamine hydrochloride at a dose of 100 mg and fentanyl citrate at a dose of 3 μg/kg intravenously. General anesthetic was used by continuous infusion pentobarbital (5 to 6 mg/kg/h) or combination of propofol (75 to 150 mg/kg/h) in supine position [14], and was adjusted upward as tolerance developed. Paralysis was achieved with Pancuronium (bolus = 0.16 mg /kg, followed by 0.08 mg /kg/ h) [15]. Orotracheal intubation was performed using a cuff tube of 8.0mmID, and lungs were ventilated using EVITA_4_ ventilator ( Dräger Medical AG,Germany). A/C-V mode was first adopted. The tidal volume (VT) was set to 10 ml/kg. PEEP was 5 cmH_2_O, I:E ratio 1:1, and FiO_2_ 1.0, RR (respiratory rate) was regulated, thus keeping PaCO_2_ between 35 and 45 mmHg. The femoral artery and the jugular vein on the right were catheterized to the PiCCO system (Pulsion Medical Systems, Munich, Germany) to measure the average arterial pressure and the core temperature. A catheter combined with multiple pairs of esophageal balloon electrodes (Guangzhou Yinghui Medical Technology Co. Ltd, China) was inserted into esophagus. The appropriate position was checked using airway occlusion technique [16]. Gastric pressure (Pgas)、airway pressure (Paw)、 esophageal pressure (Peso) 、electromyography of diaphragmatic esophagus（EMGdi）and Electromyography of abdominal muscle (EMGab) were recorded by PowerLab 16/30SP and Chart7.2 software (ADInstruments,Ltd,Australia). A respiratory flow head (MLT300L) was adopted to measure the airflow. Body temperature was kept constant at 37 ℃ throughout the experiment using an electric warming pad. Lactated Ringer^’^s intravenous fluid was injected at a rate of 6 ml/kg/h to keep the average arterial pressure as 70 mmHg. PiCCO calibrated after 8 h by transpulmonary thermodilution.

### Experiment protocol

Respiratory mechanics data of beagle dogs were measured after 30 min., and then,a total of 0.2 ml/kg purified Oleic Acid (OA)was injected to induce lung injury, if needed, additional infusion oleic acid (0.1 ml each time) would be given. Until PaO_2_/FiO_2_ were consistently between 100 to 300 mmHg for 30 min, a stable model of mild or moderate ARDS was considered to be established successfully [17–19]. After lung injury, the ventilator mode was switched to the BIPAP mode. Beagles were split into (1) SB group (BIPAP_SB_ group) and (2) Complete muscle paralysis group (BIPAP_PC_ group). In the BIPAP_PC_ group, the P_high_ was regulated, thus keeping the VT around 6 ml/kg. P_low_ was pre-set to 10 cm H_2_O, FiO_2_ at 1.0, and I:E at 1:1. RR were regulated to keep the level of PaCO_2_ between 45 and 60 mmHg. In BIPAP_SB_ group, the infusion of pancuronium bromide was ceased, and the dosage of pentobarbital and propofol decreased gradually to recover SB, and other ventilator settings were identical to those of BIPAP_PC_ group. The control group was only induced by OA. After 8 h ventilation, all the animals were euthanized through venous infusion of potassium chloride. Lung tissue samples were collected from the upper lobes, the latera lobes, the dorsal and ventral parts of the lower lobe of the right lung, respectively, and then placed in 10% buffered formalin for the subsequent histological analysis. Tis experiment was carried out by observing the “Guidelines for the Care and Use of Laboratory Animals” (NIH Publication No. 85–23, 2011) published by the National Institutes of Health. All of the animal procedures are approved by the Animal Experimental Ethical Inspection Form of Guizhou Medical University (approve number: 1603175) and carried out in compliance with the ARRIVE guidelines.

### Measurements of respiratory mechanics, EELV and VD/VT

All the variables were constantly recorded by PowerLab. For BIPAP mode, the following equation could be adopted to calculate the mean airway pressure (mean Paw) [20]: (P_high_ × T_high_ + P_low_ × T_low_) / (T_high_ + T_low_), where T_high_ denotes the length of time for P_high_, and T_low_ that for P_low_. If T_high_: T_low_ was set to 1:1, the mean value of Paw could be remained constant even though the RR changes. By regulating the ventilator using the method mentioned above, a comparable mean Paw level could be kept in this study. The transpulmonary pressure (P_L_) was calculated by the equation: P_L_ = Paw–Peso. The peak airway pressure (Ppeak) was recorded, and the total RR was calculated by the swings of Pes. End-expiratory volume (EELV) was ascertained using a simplified closed-circuit helium dilution method [21]. The alveolar dead space-to-tidal volume ratio (VD/VT) was calculated by: VD/VT = PaCO_2_- ETCO_2_/ PaCO_2_ [22].

### Inflammatory mediators

The lower lobes on the left were lavaged with 40 ml sterilized normal saline and recycled after 5 s. Plasma was gathered before the induction of ARDS, during the injury as well as in the end of the experiment. These plasma samples and Bronchoalveolar lavage fluid (BALF) were centrifuged at a rate of 3,000–4,000 rpm at once for 15 min. The protein levels of IL-6 and IL-8 were measured with enzyme-linked immunosorbent assay kit specially made for dogs (Genequick, Guangzhou, China). The expression levels of the mRNA of IL-6 and IL-8 were ascertained through the quantitative real-time reverse-transcription polymerase chain reaction. Glyceraldehyde-3-phosphate dehydrogenase (GAPDH) primers were adopted as the internal control for the normalization of RNA template. The senses and anti-senses of the taken primers (5’-3’) for IL-6 and -8 were presented as follows:

- IL-6 F: TGACCACTCCTGACCCAACC, R: TCCAGACTCCGCAGGATGAG;
- IL-8 F: ACTTCCAAGCTGGCTGTTGC, R: CTGGCATCGAAGTTCTGAACTG.

### Histopathological examination

The animals were sacrificed by 100 mg/kg of intravenous 10% potassium chloride [23]. Biopsies were collected on the middle lobes, upper lobes, and lateral, ventral and dorsal parts of the right lower lobe, respectively. Subsequently, they were placed into 10% buffered formaldehyde and then stained with HE (hematoxylin–eosin). A pathologist was appointed to examine all the collected biopsies with the lung injury histopathology scoring system. The scoring system included: 1, none; 2, mild; 3, moderate; 4, severe. The following criteria for each level: alveolar and interstitial edema, granulocytes, lymphocytes and erythrocytes infiltrate, fibrinous exudates and micro thrombi. The total score was obtained by up-regulating all the sub-scores [24].

### Statistical analysis

All the dates are expressed as the means ± SDs. The data of hemodynamics and respiratory mechanics between the two experimental groups were compared by unpaired t-test, and paired t-test was used to evaluate the difference before and after modeling in the same group. The differences of EELV, VD/VT, Pao2/FiO2 and inflammatory factors between groups were analyzed by one-way ANOVA and post-test by LSD-t or Dunn’s procedure. The changes of hemodynamics and respiratory mechanics parameters affected by intergroup and time were measured by double analysis of variance (ANOVA). Differences were considered statistically significant if P value was below 0.05. GraphPad Prism 8. 3 and SPSS21.0 software were used to drawing and perform statistical analyses. The power analysis was calculated by analyzing the final pathological injury score of lung tissue, inflammatory factors and oxygenation index of two groups of beagle dogs.GPower3.1 were used to calculate power analysis.

## Results

There was no significant difference in the basic data (weight, length and age, or the dosage of OA injection) in the experimental groups. Hypoxia and acidosis appeared, and respiratory system static compliance decreased significantly after the infusion of OA.

### Hemodynamics and gas exchanges

#### Hemodynamics

Table 1 suggests that there was no difference in the hemodynamic parameters at the beginning and the end of the experiment.

**Table 1:** Hemodynamics and respiratory measurements

Variables	Group (*n* = 6)	Before ARDS	After Induction of ARDS					Group Effect	Time Group Effect
			**injury**	**2 h**	**4 h**	**6 h**	**8 h**		
**Mean arterial**	**BIPAP**_**SB**_	**100 ± 16**	**118 ± 15**	**112 ± 13**	**116 ± 12**	**118 ± 22**	**119 ± 18**	**0.886**	**0.724**
**pressure (mmHg)**	**BIPAP**_**PC**_	**103 ± 12**	**110 ± 21**	**115 ± 17**	**117 ± 20**	**116 ± 11**	**114 ± 16**		
**Heart rate**	**BIPAP**_**SB**_	**137 ± 15**	**134 ± 21**	**131 ± 23**	**129 ± 14**	**126 ± 21**	**129 ± 14**	**0.532**	**0.478**
**(beats/min)**	**BIPAP**_**PC**_	**139 ± 13**	**137 ± 23**	**133 ± 21**	**123 ± 15**	**122 ± 17**	**128 ± 17**		
**CI**	**BIPAP**_**SB**_	**4.7 ± 0.5**	**3.7 ± 0.7**	**3.2 ± 0.4**	**3.8 ± 0.7**	**3.1 ± 0.3**	**3.4 ± 0.4**	**0.385**	**0.542**
**(L/min/m2)**	**BIPAP**_**PC**_	**4.3 ± 0.4**	**3.9 ± 0.6**	**3.5 ± 0.4**	**4.0 ± 0.5**	**3.2 ± 0.3**	**3.6 ± 0.4**		
**CO**	**BIPAP**_**SB**_	**2.4 ± 0.2**	**1.7 ± 0.4**	**1.5 ± 0.3**	**1.8 ± 0.3**	**1.7. ± 0.3**	**1.9 ± 0.5**	**0.556**	**0.815**
**(L/min/m2)**	**BIPAP**_**PC**_	**2.2 ± 0.4**	**1.6 ± 0.3**	**1.7 ± 0.4**	**1.7 ± 0.3**	**1.7 ± 0.4**	**1.8 ± 0.4**		
**PH**	**BIPAP**_**SB**_	**7.38 ± 0.09**	**7.25 ± 0.15**	**7.24 ± 0.17**	**7.22 ± 0.27**	**7.21 ± 0.28**	**7.21 ± 0.21**	**0.731**	**0.632**
	**BIPAP**_**PC**_	**7.37 ± 0.07**	**7.23 ± 0.17**	**7.24 ± 0.10**	**7.23 ± 0.15**	**7.22 ± 0.26**	**7.20 ± 0.29**		
**PaO2/FiO2**	**BIPAP**_**SB**_	**412 ± 46**	**176 ± 62**	258 ± 54*#	294 ± 74*#	343 ± 69*#	379 ± 70*#	**0.025**	**0.031**
**(mmHg)**	**BIPAP**_**PC**_	**423 ± 51**	**185 ± 55**	205 ± 49*#	224 ± 65*#	254 ± 73*#	278 ± 69*#		
**PaCO2**	**BIPAP**_**SB**_	**49.3 ± 9.6**	**58.9 ± 10.6**	**53.6 ± 7.3**	**45.2 ± 4.6**	**47.1 ± 7.8**	**55.6 ± 5.9**	**0.712**	**0.432**
**(mmHg)**	**BIPAP**_**PC**_	**48.2 ± 8.1**	**59.4 ± 12.4**	**54.5 ± 5.8**	**48.2 ± 7.8**	**51.6 ± 9.1**	**57.5 ± 7.8**		
**Total RR**	**BIPAP**_**SB**_	**22 ± 6**	35 ± 3*#	36 ± 4*#	36 ± 5*#	34 ± 4*#	32 ± 3*#	**0.0001**	**0.007**
**(Bpm)**	**BIPAP**_**PC**_	**21 ± 5**	44 ± 6*#	48 ± 5*#	43 ± 5*#	46 ± 8*#	47 ± 6*#		
**VTave**	**BIPAP**_**SB**_	**10.2 ± 0.3**	**6.4 ± 1.4**	**6.7 ± 1.4**	**6.4 ± 2.1**	**6.7 ± 1.5**	**6.5 ± 1.6**	**0.276**	**0.96**
**(ml/kg)**	**BIPAP**_**PC**_	**10.1 ± 0.2**	**6.7 ± 0.9**	**6.7 ± 0.6**	**7.0 ± 0.7**	**7.1 ± 0.8**	**7.1 ± 0.7**		
**Plateau**	**BIPAP**_**SB**_	**6.4 ± 1.1**	**20.2 ± 1.4**	**20.3 ± 0.6**	**20.6 ± 0.8**	**20.8 ± 0.7**	**21.2 ± 1.2**	**0.684**	**0.783**
**Pressure (cmH2O)**	**BIPAP**_**PC**_	**6.5 ± 1.3**	**20.1 ± 1.0**	**20.4 ± 0.9**	**20.7 ± 0.7**	**20.8 ± 0.6**	**21.4 ± 1.5**		
**Mean airway**	**BIPAP**_**SB**_	**8.4 ± 0.5**	**15.2 ± 1.4**	**15.0 ± 0.6**	**15.5 ± 0.8**	**15.4 ± 0.7**	**15.1 ± 0.9**	**0.516**	**0.908**
**Pressure (cmH2O)**	**BIPAP**_**PC**_	**8.5 ± 0.3**	**15.1 ± 1.0**	**15.7 ± 0.9**	**15.4 ± 0.7**	**15.8 ± 0.6**	**15.9 ± 0.8**		
**Peak Transpulmonary**	**BIPAP**_**SB**_	**5.4 ± 0.5**	**15.2 ± 4.4**	22.0 ± 0.6*	23.5 ± 1.1*	23.3 ± 1.2*	23.7 ± 1.1*	**0.632**	**0.01**
**Pressure(cm H2O)**	**BIPAP**_**PC**_	**5.5 ± 0.3**	**14.1 ± 3.0**	20.7 ± 0.5 *	21.4 ± 0.7*	20.8 ± 0.6*	20.5 ± 0.8*		
**MeanTranspulmonary**	**BIPAP**_**SB**_	**5.4 ± 0.5**	**15.2 ± 1.3**	15.0 ± 0.6*	14.5 ± 0.7*	15.3 ± 0.8*	15.7 ± 0.6*	**0.562**	**0.01**
**Pressure(cm H2O)**	**BIPAP**_**PC**_	**5.5 ± 0.3**	**14.1 ± 1.2**	17.7 ± 0.5*	18.4 ± 0.8*	17.8 ± 0.7*	17.5 ± 0.8*		
**Expiratory intragastric**	**BIPAP**_**SB**_	**—**	**4.1 ± 0.8**	**—**	**—**	**—**	9.5 ± 1.1*#	**0.02**	**0.00**
**Pressure (cm H2O)**	**BIPAP**_**PC**_	**—**	**4.1 ± 1.1**	**—**	**—**	**—**	5.1 ± 0.8*		
**Pgas**	**BIPAP**_**SB**_	**—**	13.2 ± 2.6*	**—**	**—**	**—**	**—**	**0.0001**	**—**
	**BIPAP**_**PC**_	**—**	5.3 ± 1.4*	**—**	**—**	**—**	**—**		
**ΔPes**	**BIPAP**_**SB**_	**—**	13.6 ± 1.8*	**—**	**—**	**—**	**—**	**0.0001**	**—**
	**BIPAP**_**PC**_	**—**	4.7 ± 0.7*	**—**	**—**	**—**	**—**		
**Lung compliance**	**BIPAP**_**SB**_	**1.9 ± 0.2**	**0.4 ± 0.1#**	**—**	**—**	**—**	**—**	**—**	**—**
	**BIPAP**_**PC**_	**1.8 ± 0.1**	**0.5 ± 0.1#**	**—**	**—**	**—**	**—**		
**EELV**	**BIPAP**_**SB**_	**498 ± 52**	**316 ± 32**	258 ± 54*#	294 ± 74*#	343 ± 69*#	452 ± 42*	**0.0001**	**0.06**
**(mmHg)**	**BIPAP**_**PC**_	**496 ± 49**	**314 ± 35**	205 ± 49*#	224 ± 65*#	254 ± 73*#	332 ± 37*		
**VD/VT**	**BIPAP**_**SB**_	**29 ± 2.7**	62 ± 4.9*#	52 ± 5.6*#	48 ± 4.4*#	44 ± 5.7*#	42 ± 4.3*#	**0.0001**	**0.0001**
	**BIPAP**_**PC**_	**24 ± 3.2**	58 ± 5.3*#	60 ± 5.2*#	58 ± 4.2*#	59 ± 3.8*#	57 ± 4.8*#		

Values are means ± SD. *P < 0.05 BIPAP_SB_ vs. BIPAP_PC_ group at the same time. ^#^*P* < 0.05 vs. injury in the same group

*BIPAP*_*SB*_ Biphasic positive airway pressure with SB, *BIPAP*_*PC*_ Biphasic positive airway pressure with muscles paralysis, *SB* Spontaneous Breathing, *NS* No Significantly Difference, *SB* Spontaneous Breathing, *PC* Complete muscle paralysis, *MVtot* Total Minute Ventilation, *CI* cardiac index, *CO* cardiac output, *PaCO*_*2*_ Partial pressure of carbon dioxide, *PaO*_*2*_*/FiO*_*2*_ Ratio of partial pressure of arterial oxygen to faction of inspired oxygen concentration

#### Respiratory mechanics

**Fig. 1:**
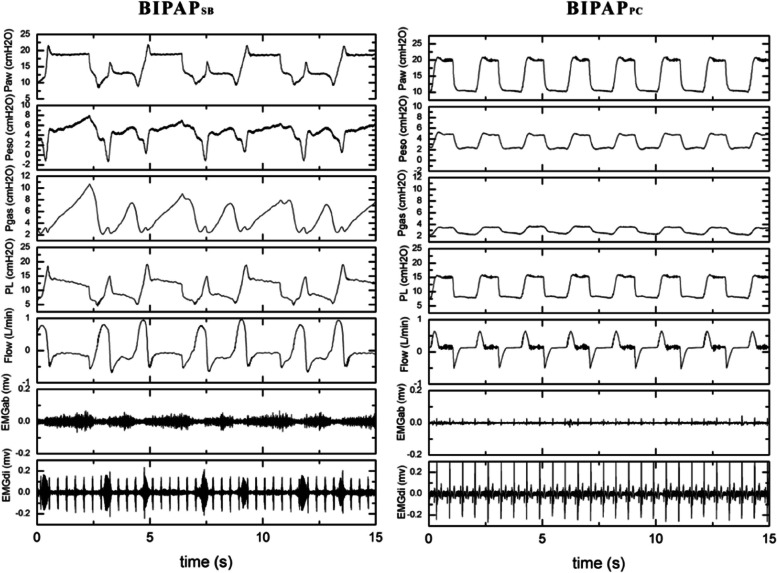
Representative respiratory tracings of airway pressure (Paw), esophageal pressure (Pes), intragastric pressure (Pgas), transpumonary pressure (PL), Airflow, abdominal muscles surface electromyography (EMGab) and diaphragmatic esophageal surface electromyography (EMGdi) in BIPAP, BIPAP group in representative animals. BIPAP_SB_ = biphasic positive airway pressure with spontaneous breathing, SB efforts were regained; BIPAP_PC_ = biphasic positive airway pressure with muscles paralysis, Animals’ SB efforts were fully depressed. Therefore, BIPAP was equal to pressure-controlled ventilation

Figure 1 and Table 1 suggest that there was also a comparable mean Paw between the two experimental groups throughout the experiments. The pressure–time curve showed that spontaneous breathing appeared in BIPAP_SB_ group, and spontaneous breathing mainly occurred in low pressure. In BIPAP_PC_ group, there was no spontaneous breathing, which was a typical curve of pressure controlled ventilation. Compared with BIPAP_PC_, the EMGdi of BIPAP_SB_ group was visible. Due to the diaphragm activity, BIPAP_SB_ group exhibited higher peak P_L_, Pes, and Pgas and lower mean transpulmonary pressure compared with BIPAP_PC_. BIPAP_PC_ group had neither diaphragmatic activity nor abdominal muscle activity. As a result, its Peso experienced a positive variation in the inspiratory phase. In addition, After 6 h of modeling, the respiratory rate in BIPAP_PC_ was significantly higher than that in BIPAP_SB_, but there was no significant difference in tidal volume between the two groups.

#### EELV

**Fig. 2:**
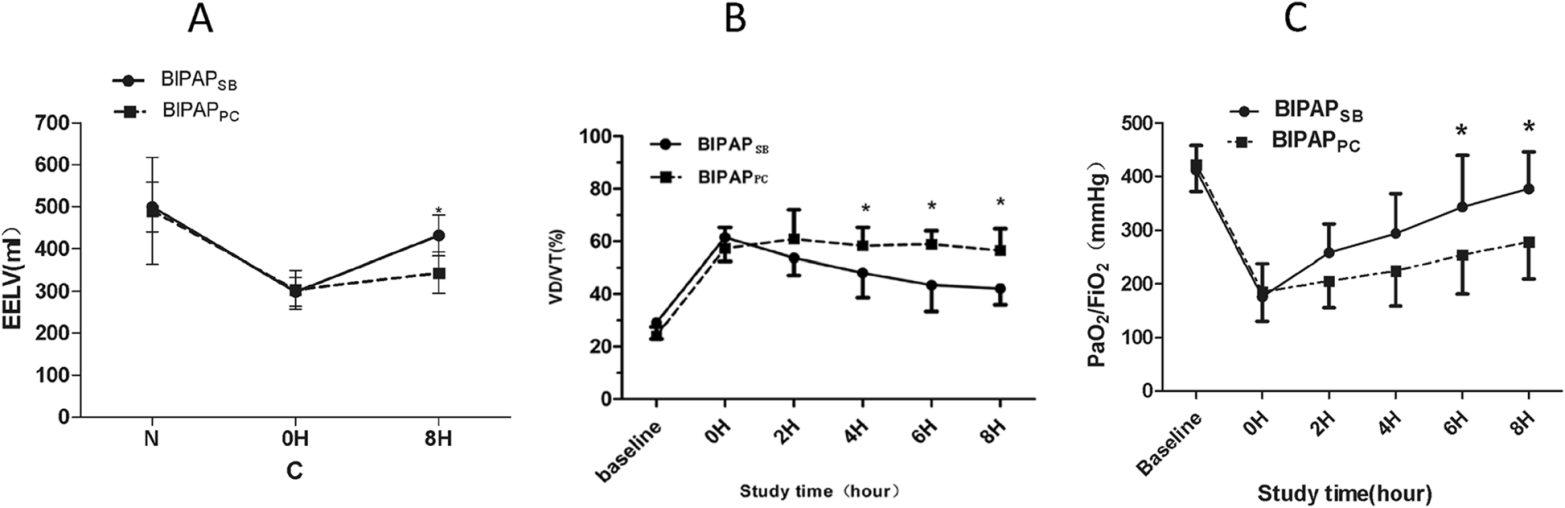
**A** Time course of the end- expiratory lung volume (EELV). **B** Time course of the dead space volume to tidal volume (VD/VT) ratio. **C** Time course of the oxygenation index.BIPAP_SB_ = Biphasic positive airway pressure with SB; BIPAP_PC_ = Biphasic positive airway pressure with muscles paralysis; SB = Sspontaneous Breathing; *P < 0.05, vs. other groups

Figure 2A suggests that the EELV decreased after the induction of lung injury, and no difference was detected in these groups at the beginning of the ventilation. After the planned MV strategy was adopted, the experimental groups showed an overt difference in EELV after 8 h of ventilation (*P* < 0.001). The EELV of BIPAP_SB_ group was higher than that of BIPAP_PC_ group (*P* < 0.05).

#### VD/VT

Figure 2B shows no significant difference in VD/VT before and after the induction of lung injury. After the planned MV strategy was adopted, the experimental groups showed a significantly difference in VD/VT after 8 h of ventilation (*P* < 0.05). BIPAP_SB_ group (50.6 ± 6.7%) led to a lower VD/VT than BIPAP_PC_ group after 2 h of ventilation, but a significant difference was also found after 6 h of ventilation (*P* < 0.05).

#### Gas exchanges

As shown in Fig. 2C, there was no significant difference in P/F before and after lung injury. The P/F of all the beagle dogs decreased between 100 to 200 mmHg after injection of OA. After the planned MV strategy was adopted, BIPAP_SB_ group showed a higher P/F than that of the BIPAP_PC_ group after 6 h of ventilation (*P* < 0.05).

#### Lung and systemic inflammatory mediators

**Fig. 3:**
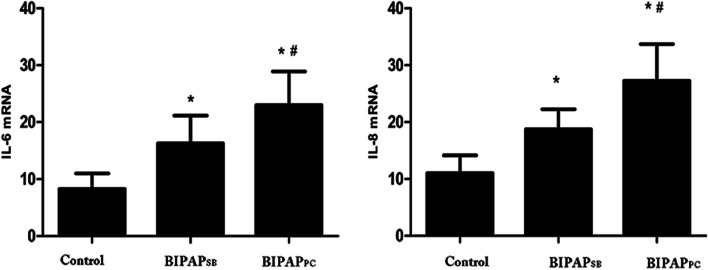
The Levels of interleukin (IL)-6 and IL-8 in plasma after 8 h mechanical ventilation(*P < 0.05 vs. other groups).Control = Control group;BIPAP_SB_ = Biphasic positive airway pressure with SB; BIPAP_PC_ = Biphasic positive airway pressure with muscles paralysis SB = Spontaneous Breathing; NS = No Significantly Difference

As displayed in Fig. 3, the IL-6 and IL-8 levels in the plasma were comparable among groups after the induction of lung injury. Nevertheless, BIPAP_SB_ group yielded lower IL-8 levels compared with BIPAP_PC_ (*P* < 0.05); Moreover, BIPAP_SB_ group exhibited lower mRNA expression levels of IL-6 and IL-8 compared with BIPAP_PC_ (*P* < 0.05), whereas all were higher than control group.

#### Lung histopathology

**Fig. 4:**
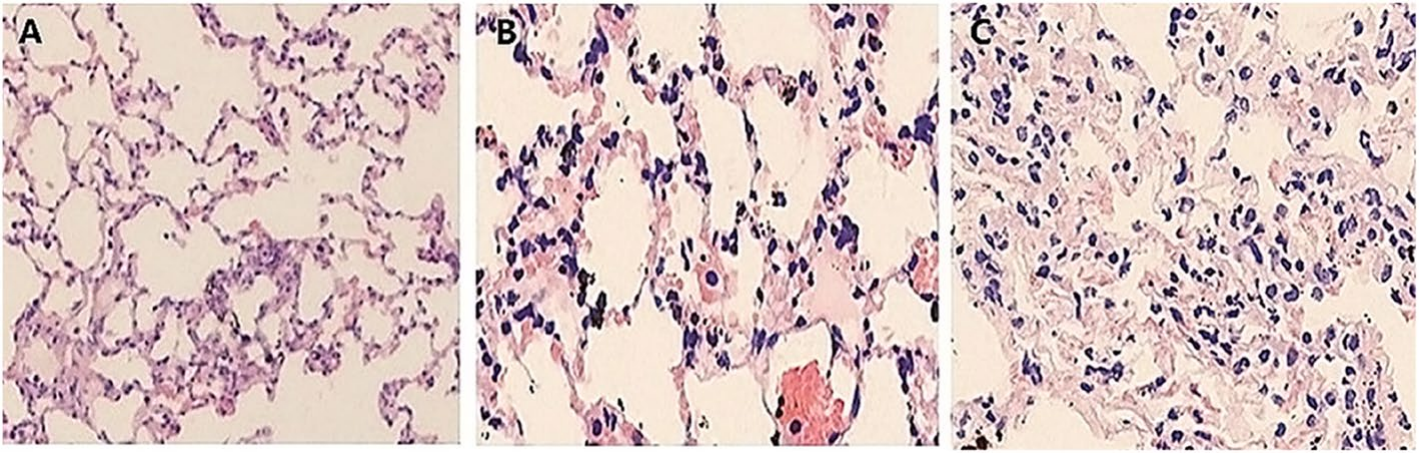
Representative appearances and photomicrographs of hematoxylineosin–stained lung sections (magnification × 200) from in Control (**A**), BIPAP_SB _(**B**), BIPAP_PC_ (**C**) group in representative animals. BIPAP_SB_ = Biphasic positive airway pressure with SB; BIPAP_PC_ = Biphasic positive airway pressure with muscles paralysis. The BIPAP _SB_ group had minimal alveolar congestion, and inflammatory cell infiltration. The BIPAP_PC_ group showed mild thickening of the alveolar walls, alveolar congestion, and hemorrhage

Table 2 and Fig. 4 suggest that the overall cumulative histopathological lung injury score of BIPAP_SB_ group was lower than that of BIPAP_PC_ group, but all were higher than that of control group. BIPAP_SB_ group presented less lung congestion, pulmonary edema, alveolar neutrophils infiltration and interstitial lymphocyte infiltration. In the meantime, BIPAP_PC_ group took on more alveolar rupture, inflammatory cell infiltration, and alveolar congestion, as well as thicker alveolar wall and greater interstitial edema accompanied with the formation of hyaline membrane.

**Table 2:** Histological subscores in experimental groups

	**Control**	**BIPAP**_**SB**_	**BIPAP**_**PC**_	***P* value**
**Congestion**	1.1 ± 0.5	1.8 ± 0.5	2.6 ± 0.7	0.006
**Edema, interstitial**	1.2 ± 0.5	1.4 ± 0.5	1.2 ± 0.4	0.689
**Edema, alveolar**	1.1 ± 0.7	2.1 ± 0.7	3.3 ± 0.5	0.001
**Granulocyte infiltrate, interstitial**	1.5 ± 0.6	2.5 ± 0.6	2.8 ± 0.6	0.019
**Granulocyte infiltrate, alveolar**	1.0 ± 0.6	2.0 ± 0.6	2.1 ± 0.5	0.813
**Erythrocyte infiltrate, interstitial**	1.6 ± 0.4	1.8 ± 0.4	2.8 ± 0.6	0.006
**Erythrocyte infiltrate, alveolar**	1.4 ± 0.6	2.8 ± 0.6	2.9 ± 0.5	0.014
**Lymphocyte infiltrate, interstitial**	1.2 ± 0.5	1.2 ± 0.5	1.0 ± 0.3	0.238
**Microthrombi**	1.2 ± 0.3	2.2 ± 0.3	2.5 ± 0.4	0.156
**Fibrinous exudate, interstitia**	1.3 ± 0.5	2.3 ± 0.5	2.5 ± 0.5	0.027
**Fibrinous exudate, alveolar**	1.2 ± 0.3	1.2 ± 0.3	1.7 ± 0.5	0.185
**Cumulative score**	15 ± 1.8	21.1 ± 2.1	24.8 ± 2.3	0.003

**Values are means ± SD**

*BIPAP*_*SB*_ Biphasic positive airway pressure with SB, *BIPAP*_*PC*_ Biphasic positive airway pressure with muscles paralysis, *SB* Spontaneous Breathing, *PC* Complete muscle paralysis, Grading as: 0, minimal changes; 1, mild; 2, moderate; 3, severe; 4, maximal changes

## Discussion

The mechanical ventilation is required to support gas exchange in the ARDS, whereas it may aggravate lung damage, a phenomenon known as ventilator-induced lung injury (VILI). Spontaneous breathing and complete muscle paralysis are two treatment methods of ARDS mechanical ventilation. but it is currently in dispute with the role of spontaneous breathing in ARDS ventilation. It is now widely considered that maintaining spontaneous breathing may have different physiological effects in mild, moderate and severe ARDS,In severe ARDS, SB could aggravate lung injury, whereas in animal with mild or moderate ARDS, SB might be more protective for injured lung,For patients with severe ARDS, excessive spontaneous breathing will lead to increased transpulmonary pressure, lung gas swing, pulmonary edema and man–machine asynchrony, which will lead to the aggravation of lung injury and increase the mortality of patients;For patients with mild to moderate ARDS, spontaneous breathing may improve alveolar ventilation and oxygenation in gravity-dependent areas by increasing diaphragm activity [7, 13]. However, few research has clarified the mechanical mechanism of SB on oxygenation and lung injury in mild or moderate ARDS. The present study is the first to demonstrate the mechanism of SB improved respiratory function and mitigated VILI in mild or moderate ARDS.

Based on an OA-induced ARDS model in beagles, we found SB improved respiratory function and mitigated lung injury, which is consistent with the previous animal and clinical experiments [11, 25–27]. In our study, we found that BIPAP_SB_ group presented a higher EELV than BIPAPpc group, as well as a lower VD/VT than BIPAP_pc_ group in the same mean airway pressure. Those two research results could explain mechanism why preserving SB improved respiratory function. Spontaneous inspiratory preserving diaphragm muscle contraction result in a higher and safety Peak P_L,_ which would promote the dorsal-caudal distribution of ventilation, thereby increasing EELV. The factors contribute to more aeration in dependent lung regions, so as to improve gas exchange. Douglas et al. [28] have also proven that the increase in EELV was parallel to oxygenation. Furthermore, preserving SB presents lower VD/VT, which means a more appropriate ventilation-perfusion matching, as well as an improved respiratory function [8].

In this study, it was also found that the lung mRNA expressions of IL-6 and IL-8, VD/VT, and lung histopathological score were lower in BIPAP_SB_ group than those in BIPAP_PC_ group, which was consistent with other study results [29], whereas few studies have elucidated the mechanism. Several mechanical mechanisms observed in our study can explain these phenomena. Firstly, In SB group, inspiratory and expiratory muscle were all retained. As soon as spontaneous inspiratory induced the ventilation, inspiratory activity would be reflex suppressed due to mechanical inflation. Ventilator could activate the expiratory muscles, especially abdominal muscles, as can be seen from the esophagus pressures and intragastric, representing the intrathoracic pressures and intra-abdominal, respectively. Our study found that the mean transpulmonary pressure in SB group was significantly lower than that in BIPAP_PC_ group, while intra-abdominal pressure was significantly higher than that in BIPAP_PC_ group. This was because spontaneous expiration unopposed increased the intra-abdominal pressure and reduced the mean transpulmonary pressure. The major factors of mechanical damage were peak transpulmonary pressure and mean transpulmonary pressure. Accordingly, by increasing intra-abdominal pressure and decreasing the mean pulmonary pressure, spontaneous abdominal muscles activity would cause less VILI in mild or moderate ARDS. On the other hand,SB up-regulates peak transpulmonary pressure led to more aeration for dorsal lung tissue, recruits dependent lung tissues that were less aerated, and decreased the repeat opening and closing cycle of lung tissue, thereby mitigating VILI.Second,strain( tidal volume/end-expiratory volume) is one of the major determinants of VILI,In this experiment, compared with completely controlled ventilation, the average VT of spontaneous breathing group decreased, while EELV increased, so the strain value decreased relatively [30]. Third, compared with BIPAP_PC_, tidal volume in SB group was variable. Studies have shown that variable tidal volume has a protective effect on VILI [31]. Last,SB may help to alleviate Ventilator induced diaphragm dysfunction(VIDD). Our study shows that Complete muscle paralysis is more relaxed than diaphragm. Previous studies have confirmed that significant diaphragm fiber atrophy occurs at 18–69 h after controlled ventilation, which leads to VIDD;VIDD is related to weaning failure and prolonged hospitalization in patients with mechanical ventilation, while keeping spontaneous breathing during mechanical ventilation can prevent diaphragmatic atrophy and effectively avoid VIDD [32]. In addition, compared with keeping SB, PC has a higher demand for sedation, analgesia and NMBA, while the use of high-dose sedation, analgesia and NMBA is related to the difficulty of weaning [33]. Therefore, proper retention of SB may help to shorten the time of mechanical ventilation and promote early activity by reducing the dose of sedation, analgesia and NMBA.

There are several major limitations in this study. Firstly, we used BIPAP ventilated mode in this study. Therefore, we are not sure whether these results can be extended to other modes. Secondly, the ARDS model induced by oleic acid cannot be extrapolated to other ARDS models. Third, because long-term ventilation time may affect the accuracy of the experiment, such as hypercapnia, drug overuse, this study used 8h ventilation observation. In fact, longer study time may lead to significant physiological and morphological differences between the experimental groups. Finally,We used the pathological injury score of lung tissue and inflammatory factors in three groups to evaluate the statistical power. There were 6 beagle dogs in each group, and the statistical power was 0.97.However, when comparing the oxygenation index of the two groups alone, our power is low, and each group may need at least 9 beagles. The number of beagles is still one of the defects of our article.

## Conclusions

To sum up, in mild to moderate ARDS animal models, SB improved respiratory function and reduced VILI. The mechanism may be that spontaneous inspiration up-regulates peak transpulmonary pressure and EELV, thus reducing strain, and spontaneous expiration decrease mean transpulmonary pressure by up-regulating intra-abdominal pressure, thereby reducing stress.

## Data Availability

The datasets used and analyzed during the current study are available from the corresponding author on reasonable request.
